# Machine Learning for Enhanced COPD Diagnosis: A Comparative Analysis of Classification Algorithms

**DOI:** 10.3390/diagnostics14242822

**Published:** 2024-12-14

**Authors:** Walaa H. Elashmawi, Adel Djellal, Alaa Sheta, Salim Surani, Sultan Aljahdali

**Affiliations:** 1 Department of Computer Science, Suez Canal University, Ismailia 41522, Egypt; 2 Department of Computer Science, Misr International University, Cairo 11828, Egypt; 3 Department of Electronics, Electrotechnics, and Automation (EEA), National Higher School of Technology and Engineering, Annaba 23000, Algeria; a.djellal@ensti-annaba.dz; 4 Computer Science Department, Southern Connecticut State University, New Haven, CT 06515, USA; shetaa1@southernct.edu; 5 Department of Pharmacy & Medicine, Texas A&M University, College Station, TX 75428, USA; surani@tamu.edu; 6 Computer Science Department, Taif University, Taif 21944, Saudi Arabia; aljahdali@tu.edu.sa

**Keywords:** chronic obstructive pulmonary disease (COPD), machine learning (ML), artificial neural network (ANN), random forest classifier (RFC)

## Abstract

**Background**: In the United States, chronic obstructive pulmonary disease (COPD) is a significant cause of mortality. As far as we know, it is a chronic, inflammatory lung condition that cuts off airflow to the lungs. Many symptoms have been reported for such a disease: breathing problems, coughing, wheezing, and mucus production. Patients with COPD might be at risk, since they are more susceptible to heart disease and lung cancer. **Methods**: This study reviews COPD diagnosis utilizing various machine learning (ML) classifiers, such as Logistic Regression (LR), Gradient Boosting Classifier (GBC), Support Vector Machine (SVM), Gaussian Naïve Bayes (GNB), Random Forest Classifier (RFC), K-Nearest Neighbors Classifier (KNC), Decision Tree (DT), and Artificial Neural Network (ANN). These models were applied to a dataset comprising 1603 patients after being referred for a pulmonary function test. **Results**: The RFC has achieved superior accuracy, reaching up to 82.06% in training and 70.47% in testing. Furthermore, it achieved a maximum F score in training and testing with an ROC value of 0.0.82. **Conclusions**: The results obtained with the utilized ML models align with previous work in the field, with accuracies ranging from 67.81% to 82.06% in training and from 66.73% to 71.46% in testing.

## 1. Introduction

**Figure 1:**
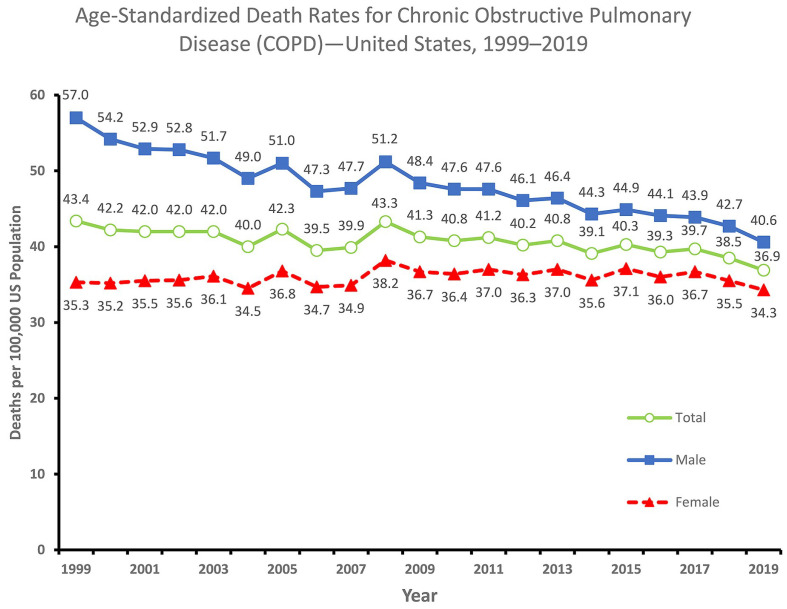
A graph of COPD age-standardized death rates by gender in the USA [1].

Chronic obstructive pulmonary disease (COPD) presents a substantial global health burden, currently ranking as the fourth leading cause of mortality in the United States. The mortality rates of the United States population are depicted in Figure 1, as reported by the National Vital Statistics System. The figure shows the age-standardized death rates for chronic obstructive pulmonary disease in the United States from 1999 to 2019. The overall death rate decreased from 43.4 deaths per 100,000 population in 1999 to 36.9 in 2019. However, the decline was not uniform across all groups. The death rate for males decreased more rapidly than the death rate for females. In 2019, the death rate for males was 40.6 deaths per 100,000 population, while the death rate for females was 34.3 deaths per 100,000 population. Overall, the figure shows that the death rate for COPD in the United States has been declining in recent years. However, there are still disparities in the death rate between males and females.

**Figure 2:**
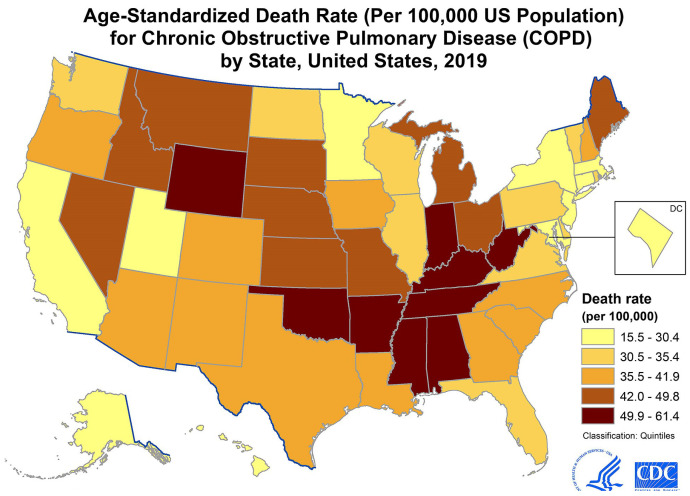
A map of the age–standardized death rate by state in the USA [1].

Figure 2 shows the age-standardized death rate by state in the USA. The color-coded map illustrates variations in death rates, with darker shades of brown indicating higher rates and lighter shades representing lower rates.

The rising cost of COPD in the United States reached USD 52 billion in 2010 (USD 32 billion in direct costs and USD 20.4 billion in indirect costs) [2]. Despite the significant educational effort, underdiagnosis and misdiagnosis remain challenges.

In terms of public health, with 3.23 million deaths in 2019, COPD ranks third among all causes of mortality globally [3]. It is a significant issue that has the potential to be handled. COPD is a global issue causing chronic morbidity and premature death, with the burden expected to rise due to an aging population and ongoing exposure to risk factors in the coming decades.

The National Institutes of Health (NIH); the National Heart, Lung, and Blood Institute (NHLBI); and other organizations collaborated to form the Global Initiative for Chronic Obstructive Lung Disease (GOLD) in 1998. Worldwide healthcare and healthcare policy communities came together as one in this endeavor to bring attention to COPD’s impact and find ways to avoid it better. Another important goal was to get more people interested in researching this prevalent disease. The Worldwide Initiative for GOLD initially published its findings in 2001 to outline the field’s present status rather than serving as a thorough textbook on COPD.

Furthermore, according to the Third National Health and Nutrition Examination Survey (NHANES III), 60% of adults with severe obstructive lung disease (OLD) do not specify that they have OLD, even though the prevalence of OLD in the USA is 15% [4,5]. Martinez et al. [6,7] also suggested that the association of low forced expiratory volume in one second ($FEV_{1}$) with mortality is a missed opportunity in diagnosis and treatment. The US Preventive Service Task Force does not suggest spirometrric screening for OLD, citing insufficient evidence for its cost-effectiveness to support the screening of the whole population.

Several societies, such as the American College of Chest Physicians, have recommended spirometry to diagnose obstructive lung disease, but it has yet to be utilized. There have also been some controversies regarding the diagnosis of OLD in terms of forced vital capacity (FVC) using an $FEV_{1}$/FVC ratio of less than 70% as cutoff, as many elderly patients may not be able to complete the FVC maneuver effectively. Several studies have suggested the use of $FEV_{1}$/$FEV_{6}$ (i.e., forced expiratory volume in 6 s) instead of the $FEV_{1}$/FVC ratio [8,9,10].

Understanding, analyzing, and extracting knowledge is becoming more complex as big data grow. More than a simple statistical study of previous decades is required in this era of continuous measurement. Finding commonalities across patients can help the healthcare industry thrive in this setting. Why, though, did we decide to address COPD and asthma exclusively? Asthma is a persistent condition that affects the airways [11]. The bronchi are the tubes that lead air to the lungs, also known as the airways. Asthmatics experience bronchospasms and airway constriction in response to environmental triggers such as inhalation. Despite a genetic predisposition, triggers, including allergens (dust, pollen, animal dander, etc.) and viral infections, cause asthma symptoms in patients. COPD is characterized by persistent shortness of breath (dyspnoea) and is often seen in patients with chronic bronchitis and emphysema. Smoking is the primary culprit in the gradual blockage of the airways. Asthma, a leading cause of mortality, affects 300 million people annually, resulting in 250,000 deaths, while COPD affects 330 million people worldwide.

Consequently, tools for the early diagnosis and prognosis of respiratory diseases must be developed to improve patient outcomes and reduce mortality rates. AI-powered techniques such as machine learning (ML) offer promising solutions. Therefore, this study aims to explore the potential of machine learning in outperforming clinicians in COPD prediction. This study investigates whether machine learning can accurately predict COPD, even without relying on spirometry. If successful, this approach promises significant cost savings through early diagnosis, facilitating targeted interventions such as smoking cessation education and risk factor mitigation. The critical contributions of this research are outlined below.

- A review of chronic obstructive pulmonary disease diagnosis models is presented.
- Various machine learning techniques are used for the diagnosis of COPD patients.
- The models are evaluated and assessed, highlighting the superior results of certain models.

### Up-to-Date Research

Given the critical impact of pulmonary diseases on human health, numerous researchers have dedicated the past decade to investigating this significant subject using machine learning. This section discusses the most recent literature on the utilization of various machine learning techniques in chronic obstructive pulmonary disease diagnosis.

One study conducted in 2017 by Amaral et al. [12] improved the accuracy of the oscillation technique (FOT) for the diagnosis of airway obstruction in asthma patients by creating classifiers that made it easier to use in the clinic, achieving an Area Under the Receiver Operating Characteristic (AUC) of up to 91%. The risk of death among hospital patients in intensive care units (ICUs) was estimated in 2018 by Darabi et al. [13] using gradient-boosted trees (GBT) and deep neural networks (DNNs). Their findings on a 10% test set demonstrated an AUC of up to 87.30%.

Zhang et al. [14] suggested a novel Cox-based learning system for failure prediction in 2019 that uses feature weighting. They came up with two weighting metrics—the AUC and the concordance index (C-index)—to boost the accuracy of the prediction. They used Dirichlet-based regularization on weights to further emphasize differences in component relevance. This helped them maintain the model’s robust predictive capabilities and achieve a sensitivity level of 72.6%.

In 2020, Moll et al. [15] explored mortality prediction through machine learning. They specifically opted for a random survival forest and utilized 30 clinical, spirometric, and imaging features as input variables for their analysis. In 2021, Nikolaou et al. [16] analyzed 6883 United Kingdom patients diagnosed with chronic obstructive pulmonary disease and at least one cardiovascular comorbidity over four years. The three cardiovascular phenotypes associated with COPD that were identified before diagnosis were successfully replicated after diagnosis with a remarkable accuracy rate of 92%. The random forest model surpasses previous models’ ability to predict hypertension and exclude less prevalent comorbidities. However, the authors of [17] used a database of yearly medical check-ups to identify the risk factors for a diagnosis of COPD using machine learning.

In [18], Meng et al. utilized five well-known supervised machine learning techniques, namely logistic regression (LR), Naïve Bayes (NB), K-Nearest Neighbors (KNN), decision tree (DT), and Random Forest (RF), to guess how likely it was that someone with COPD would have an acute exacerbation in 2022. The findings indicate that a targeted strategy involving the environmental reduction of PM2.5 emissions offers protective benefits for non-smoking COPD patients, mitigating the risk of disease exacerbation linked to air pollution.

The authors of [19] utilized a combination of Multilayer Neural Networks (MLNNs) and Extreme Gradient Boosting (XGB) to predict the prevalence of chronic obstructive pulmonary disease (COPD) in the Canadian population. The results showed an accuracy of 86% on the test dataset, which was higher than the 83% attained by the MLNN model. In [20], Wand et al. used supervised classifiers to address data imbalances in the classes. They integrated various classifier methods, such as logistic regression, Support Vector Machine (SVM), random forest (RF), XGBoost, light-GBM, NGBoost, stacking, cost-sensitive learning, and SMOTE resampling.

In 2023, primary care doctors and pulmonologists were compared to the Asthma/COPD Differentiation Classification (AC/DC) tool to determine its diagnostic accuracy [21]. The ML-based Asthma/COPD Differentiation Classification tool produced better results when used within the context of asthma, chronic obstructive pulmonary disease (COPD), and asthma-related overlap. A systematic review and meta-analysis of predictive ML models for long-term prognosis in COPD patients is presented in [22]. Consequently, the authors of [23] provided a bibliometric analysis from 2019 to 2023 on the role of AI in chronic obstructive pulmonary disease diagnosis. Furthermore, the research discussed trends and future directions.

Moreover, the authors of 2024 [24] provided a narrative review of various machine learning techniques and their applicability in the early diagnosis of COPD. Similarly, the authors of [25] reviewed the use of AI in managing COPD, focusing on how AI methods like machine learning and deep learning are used for diagnosis, treatment, and prediction. The authors of [26] aimed to create an automated tool that uses spirometry trace curvature information to diagnose COPD in primary care settings. Their study consisted of 695 participants categorized into two groups: COPD and non-COPD. The experiments demonstrated that an ANN achieved better results than SVM. Seven predictive ML models arewere employed in [27] as a simple tool for measuring COPD patients’ potential risk that may help clinicians identify high-risk patients. This study involved a performance comparison among eight distinct classifiers for COPD diagnosis. Despite the significant progress made in COPD diagnosis, there is still room for AI-driven diagnostic tools to offer promising avenues to refine COPD diagnosis and potentially revolutionize patient care.

## 2. Materials and Methods

### 2.1. COPD Dataset

**Figure 3:**
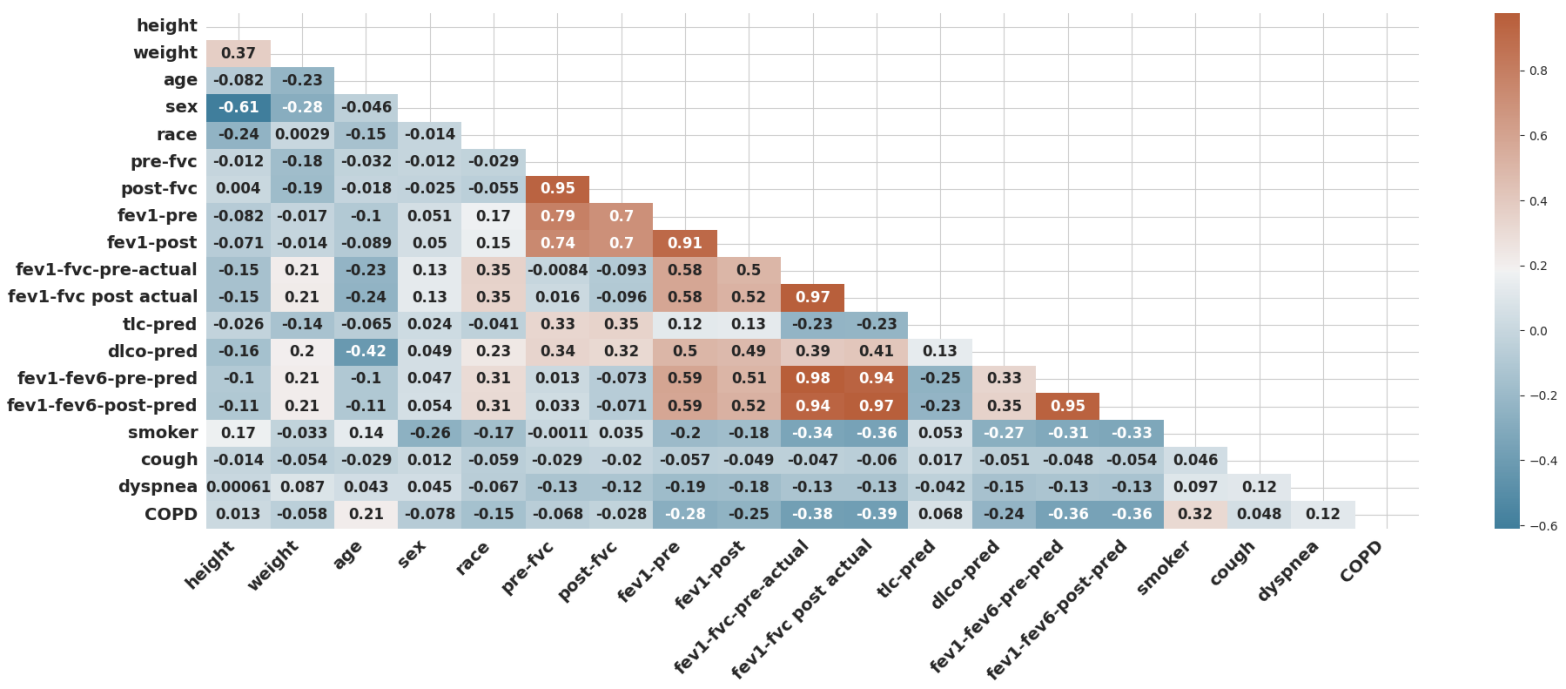
The correlation coefficients among COPD features.

The dataset used for this research was collected after receiving a waiver from the institutional review committee of Pulmonary Associates of Corpus Christi [28]. A retrospective chart review was conducted on all patients sequentially undergoing pulmonary function tests (PFTs) in a community pulmonary clinician’s office from January 2000 to June 2022. The study utilized the MGC Diagnostic product, which focuses on the Ultima PF^TM^ pulmonary function system (i.e., the MGC Diagnostics company based at 350 Oak Grove Parkway, St. Paul, MN, USA). The patients were referred for a pulmonary function test secondary to shortness of breath, possible COPD, asthma, reactive lung disease, or pre-operative clearance. The demographic dataset and pulmonary function dataset, retrieved from the Electronic Medical Records (EMRs), included demographic variables such as height, weight, age, sex, and race, as well as clinical information such as history of smoking, cough, dyspnea, and clinical COPD. The utilized dataset consists of 19 features, as shown in Figure 3.

**Figure 4:**
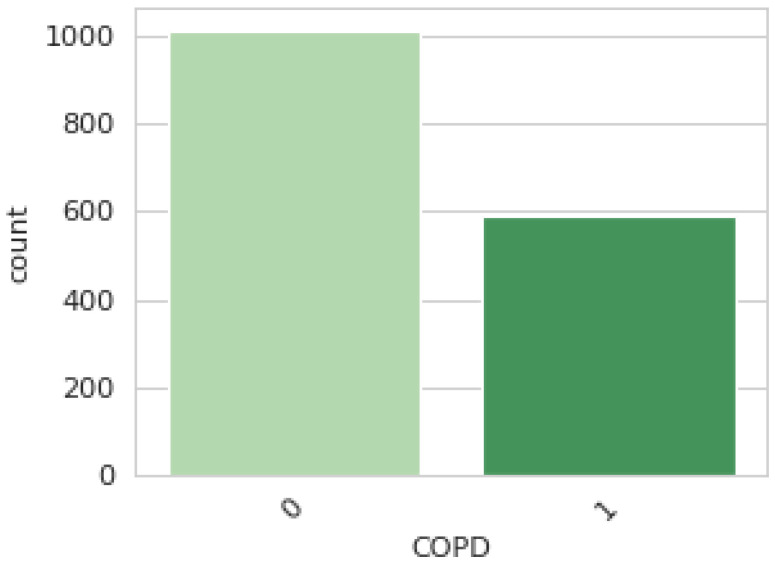
Classes (1: COPD; 0: Healthy).

As shown in Figure 4, the dataset comprises 1603 patients, comprising those diagnosed with COPD (36.68%) and healthy subjects (63.32%).

**Figure 5:**
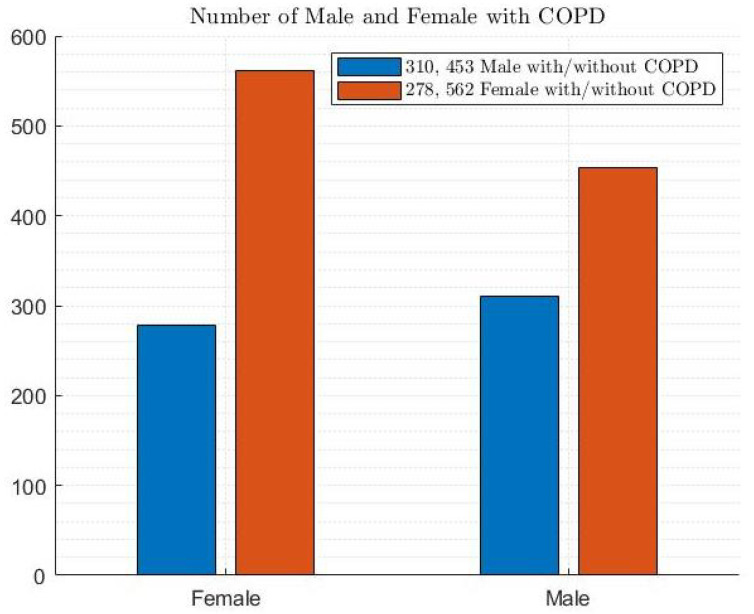
Gender distribution among patients.

Figure 5 shows the dataset with a gender distribution of 47.6% male (i.e., 310 and 453 males with and without COPD, respectively) and 52.4% female (i.e., 278 and 562 females with and without COPD, respectively).

**Figure 6:**
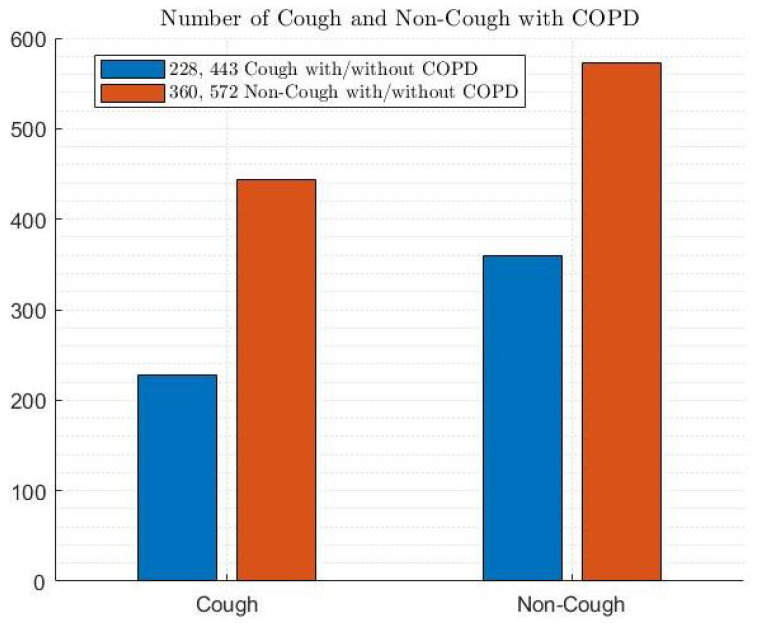
Cough patterns in patients with and without COPD.

**Figure 7:**
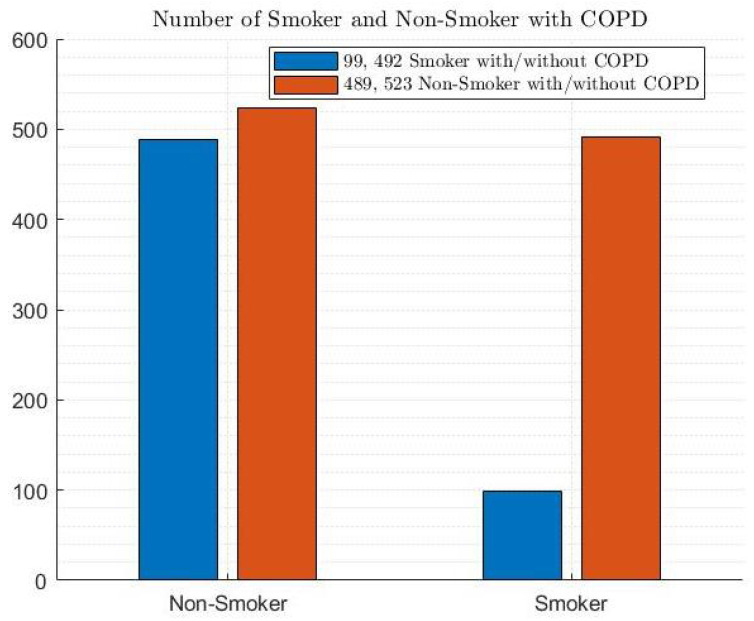
Smoking habits among patients with and without COPD.

**Figure 8:**
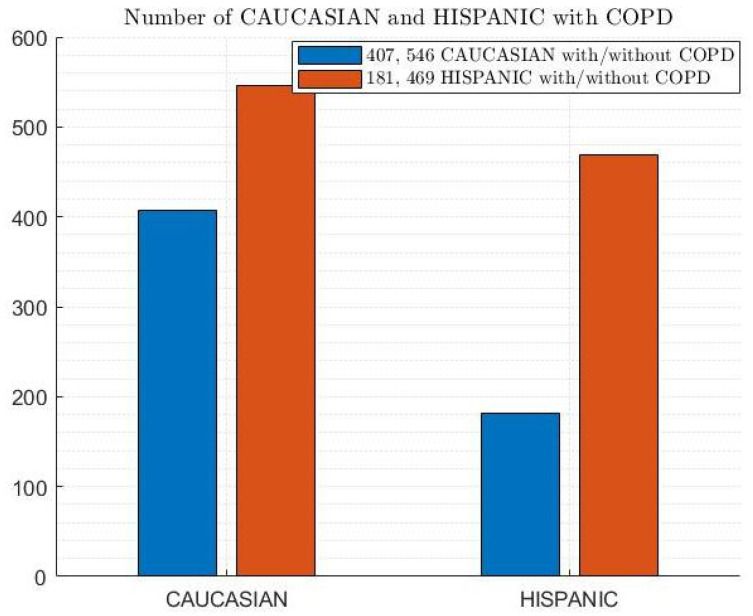
Ethnicity distribution of patients with and without COPD.

Furthermore, Figure 6, Figure 7 and Figure 8 depict the distribution of patients with and without COPD based on cough, smoking, and ethnicity, respectively. According to Figure 6, 41.9% of patients had a cough with and without COPD, while 58.1% did not have a cough with and without COPD. Regarding smoking habits, 63.1% of patients were non-smokers with and without COPD, and 36.9% of patients were smokers with and without COPD (as shown in Figure 7. In terms of the ethnic distribution of patients, Caucasians represent a higher percentage than Hispanics, with 25.4% Caucasians with COPD and 34% Caucasians without COPD and 11.3% and 29.3% Hispanics with and without COPD, respectively, according to Figure 8.

According to the standard Pulmonary Function Report, airflow obstruction was evaluated using pre- and post-bronchodilator spirometry. Lung capacity was measured using the nitrogen washout method, and diffusion capacity was rated according to the standard ATS criteria. $FEV_{1}$, FVC, $FEV_{1}/FVC$, and $FEV_{1}/FVC_{6}$ were used in the assessment before and after bronchodilator administration. In this work, several machine learning algorithms were employed to confirm the diagnosis of obstructive lung disease based on a $FEV_{1}/FVC$ ratio below 70%.

### 2.2. Machine Learning Models

Early diagnosis of COPD is crucial for effective management and improved patient outcomes by leveraging various machine learning classifier models, such as support vector machine (SVM), decision tree (DTs), random forest classifier (RFC), logistic regression (LR), Gradient Boosting Classifier (GBC), Gaussian naïve Bayes (GNB), artificial neural networks (ANNs), and K-nearest neighbors (KNN). These machine learning models are employed using the Scikit-learn library, built on Python to address our classification task. Like numerous others in the literature, this study uses respiratory data to train systems to extract essential features for diagnosis and prediction. As ML technology advances, especially in diagnosing various diseases [29,30,31,32], it holds immense promise to enhance the accuracy and accessibility of COPD screening and diagnosis, ultimately improving patients’ lives worldwide.

#### 2.2.1. Logistic Regression (LR)

**Figure 9:**
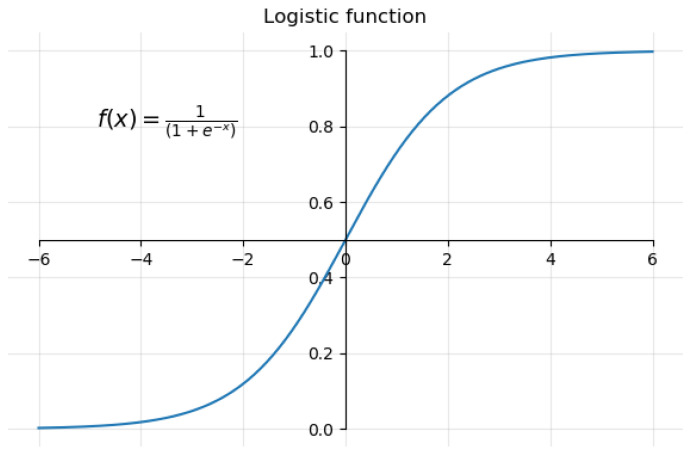
The characteristics of a logistic function.

One kind of regression analysis proposed and utilized well for binary classification is logistic regression (LR) [33,34]. Recently, it has been used in several medical applications [35]. LR utilizes predictive models to build a relationship between a dependent variable (i.e., model output) and one or more independent variables (i.e., features) by finding the best-fit logistic function ($f\left(x\right)=\frac{1}{1+e^{-(x)}}$), as shown in Figure 9.

This is achieved using Equation (1) to predict the likelihood of certain classes using the dependent variables. Based on the results of the LR model, it is ideal for binary classification problems, since its output values are consistently between 0 and 1.

(1) $$p\left(x\right)=\frac{1}{1+e^{-\left(w_{0}+w_{1}x_{1}+\cdots +w_{m}x_{m}\right)}}$$

where $w_{0}$ is a biased term, $w_{1}\ldots ,w_{m}$ are the regression coefficients (i.e., predicted weights coefficient for input *x*), and *m* is the number of independent variables (i.e., features). The LR algorithm adjusts these weights to provide maximum classification accuracy. The log-likelihood loss function can be employed for minimization. This involves negating the average log-likelihood as outlined in Equation (2).

(2) $$L=-\frac{1}{n}\sum_{n}^{i=1}y_{i}\log p\left(x_{i}\right)+\left(1-y_{i}\right)\log\left(1-p\left(x_{i}\right)\right)$$

For each observation (*i*, where i=1,2,…,n), $y_{i}$ is the label, and $p\left(x_{i}\right)$ is the predicted value of point $x_{i}$. In summary, given a set of independent variables ($x_{1},\ldots ,x_{m}$), the goal is to find the optimal parameters (*w*) such that the output ($\hat{y}$) given by Equation (3) produces a minimum cost where *X* is the array of inputs.

(3) $$\hat{y}=\sigma \left(w^{T}X+w_{0}\right)$$

#### 2.2.2. Support Vector Machine (SVM)

**Figure 10:**
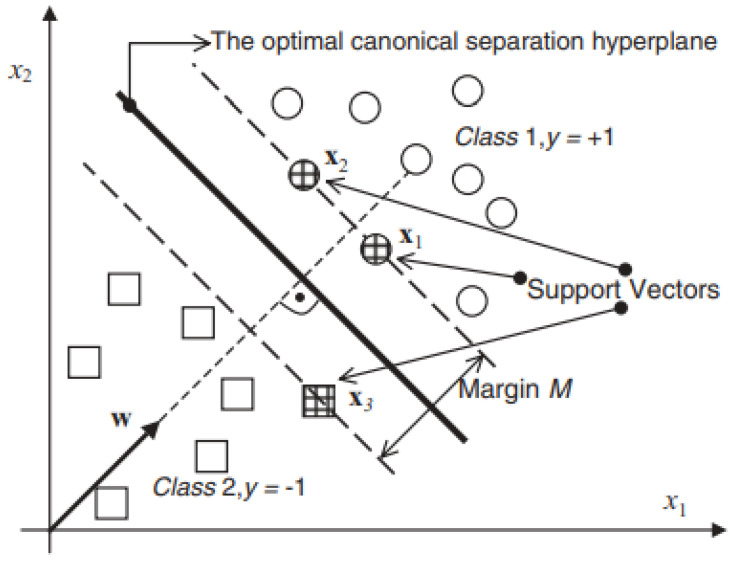
The SVM classifier and optimal separation hyperplane [37].

A Support Vector Machine (SVM) is a classification approach utilized in supervised machine learning, as stated in [36]. SVM identifies the best hyperplane for class separation by positioning the maximum number of points from the same class on one side. The SVM classifier extends the range of each class to a hyperplane, segregating the points as shown in Figure 10.

The closest points to the hyperplane are the foundation of the support vectors. The minimal distance between any two locations inside a particular class and a designated hyperplane is measured from the class to the hyperplane. In a straightforward linear separable problem, the hyperplane and SVM classifier can be delineated as per Equations (4) and (5).

(4) $$w^{T}x+b=0$$

(5) $$\hat{y}=\left\{\begin{array}{c} 1:w^{T}x+b\geq 0\\ 0:w^{T}x+b<0 \end{array}\right.$$

The variables *w*, *x*, *b*, and $\hat{y}$ stand for the weight vector, input vector, bias, and projected output class, respectively. Minimizing the ∥w∥ weight vector’s Euclidean norm is essential to optimize the margin. Hence, it can be expressed as an objective function by the following formula: $\min f:1/{2∥w∥}^{2}$.

#### 2.2.3. Gradient Boosting Classifier (GBC)

**Figure 11:**
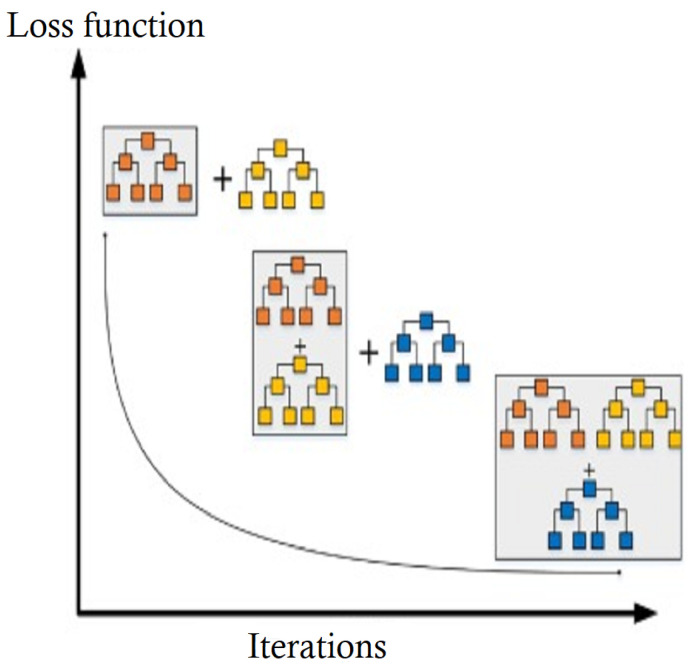
The GBC loss function across iterations [39].

As an ensemble machine learning approach, the gradient boosting classifier (GBC) combines many weak models into one more robust model, increasing the prediction power of the combined model. GBC is an efficient techniques applicable to both classification [38] and regression problems. It operates iteratively by training decision trees on the residuals of the preceding tree, utilizing gradient descent optimization to minimize the loss function as shown in Figure 11. This technique enables the algorithm to learn more intricate decision boundaries, improving prediction accuracy.

Gradient boosting is used to create an ensemble model base on a training set (D) consisting of *n* instances, where each instance has a pair of features (xi and label $y_{i}$). Several iterations (*M*), a learning rate (α), a base model ($h_{0}\left(x\right)$), and a loss function (L(y,F(x))) are required to evaluate the quality of the ensemble model, in addition to a set of hyper-parameters for the base model. The algorithm initializes the ensemble model to $F_{0}\left(x\right)=h_{0}\left(x\right)$, then iteratively improves it by fitting a base model ($h_{m}\left(x\right)$) to the negative gradient of the loss function concerning the current ensemble model (Fm−1(x)). The optimal step size ($\beta_{m}$) is computed using a line search, and the ensemble model is updated by adding a scaled version of the new base model ($h_{m}\left(x\right)$) to the previous ensemble model ($F_{m-1}\left(x\right)$). The final output of the algorithm is the resulting ensemble model (F(x)).

#### 2.2.4. Gaussian Naïve Bayes (GNB)

Naïve Bayes classifiers are machine learning algorithms rooted in the principles of Bayes’ Theorem [40] to calculate posterior probabilities. The GNB technique uses a Gaussian distribution according to Equation (6).

(6) $$P\left(C_{k}|x\right)=\frac{P\left(x|C_{k}\right)P\left(C_{k}\right)}{P(x)}$$

where $P\left(C_{k}|x\right)$ is the posterior probability, indicating the likelihood of class $C_{k}$ contingent upon the observation of *x*. The likelihood, denoted as $P\left(x|C_{k}\right)$, is the probability of observing *x* if the class is $C_{k}$. $P(C_{k})$ is the prior probability of class $C_{k}$, while P(x) is the marginal likelihood, the cumulative probability of observing *x* across all potential classes.

These classifiers rely on the assumption of solid independence among the features used for predictions. This assumption implies that the value of one feature does not influence the value of any other feature. A notable advantage of naïve Bayes classifiers is their ability to be efficiently trained in supervised learning scenarios, even when working with limited training data. Moreover, their straightforward design and ease of implementation make them popular for various real-world applications.

#### 2.2.5. K-Nearest Neighbors Classifier

One supervised machine learning technique commonly employed for classification problems is the K-nearest neighbors (KNN) classifier. It operates based on similarity, classifying unlabeled data points by considering the class of their nearest neighbors in the training dataset.

The number of neighbors to consider is represented by the “K” in KNN. The algorithm determines the distance between the unlabeled data point and all the labeled data points in the training set to make a prediction. Equation (7) can be used to calculate the Euclidean distance (ED) between two sets of data (*X* and *Y*, where $X=\left(x_{1},x_{2},\ldots ,x_{n}\right)$, $Y=\left(y_{1},y_{2},\ldots ,y_{n}\right)$, and *n* represents the features).

(7) $$Euclidean\left(X,Y\right)=\sqrt{\frac{\sum_{i=1}^{n}{\left(x_{i}-y_{i}\right)}^{2}}{n}}$$

**Figure 12:**
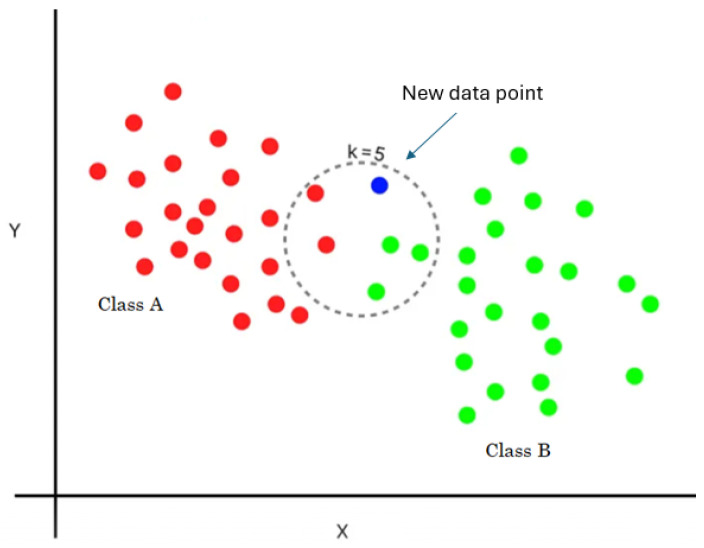
KNN classifier with K = 5.

Using these distances as a basis, it selects the K nearest neighbors. A majority vote among the K closest neighbors of the unlabeled data point determines its class identity, as shown in Figure 12. The new data point is assigned to class B (i.e., the majority class).

KNN is a simple and intuitive algorithm that does not require training, using the entire training dataset for classification.

#### 2.2.6. Decision Tree Classifier (DTC)

A robust machine learning method can be built using the data’s characteristics and specific rules: a decision tree (DT). Decision trees can be employed for many ML tasks (classification and regression) [41]. A DT learning algorithm chooses the optimal node split point. According to [42], optimal data splitting is achieved using the entropy and information gain approach. The entropy (S(Z)) determines the impurity of the sample values and is computed according to Equation (8).

(8) $$S\left(Z\right)=-\sum_{i}P\left(Z=z_{i}\right)\cdot \log_{2}\left(P\left(Z=z_{i}\right)\right)$$

where S(Z) stands for the entropy of the random variable (*Z*) and $P(Z=z_{i})$ signifies the likelihood of the event ($Z=z_{i}$).

**Figure 13:**
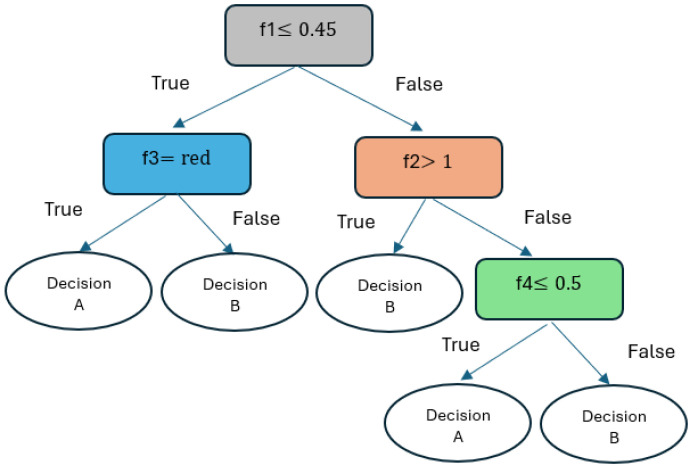
The DT classifier with four features.

Figure 13 shows a simple visualization of a DT with four features. The tree comprises root, decision, and leaf nodes (i.e., prediction nodes). Each non-leaf node pertains to a particular feature (fi) and a corresponding conditional relationship. For input instances, the decision tree follows a path to a leaf node that contains a prediction.

#### 2.2.7. Random Forest Classifier (RFC)

Random forests are collections of tree predictors that work together as an ensemble [43]. Each tree in the forest relies on values from a random vector, which is independently sampled and shares the same distribution across all trees [44]. This consists of *N* bootstrap samples from the entire dataset (*D*, i.e., $D=\{\left(f_{1},c_{1}\right),\left(f_{2},c_{2}\right),\ldots ,\left(f_{m},c_{m}\right)\}$, where $f_{i}$ represents the feature vector of the *i*-th sample and $c_{i}$ denotes the class label). The final prediction aggregates all DTs’ predictions through a majority vote in the case of classification or averaging in the case of regression. To predict the class or label of a new instance (*b*), Equation (9) is utilized.

(9) $$\hat{P}\left(b\right)=\frac{1}{T}\sum_{i=1}^{T}Q_{i}\left(x\right)$$

**Figure 14:**
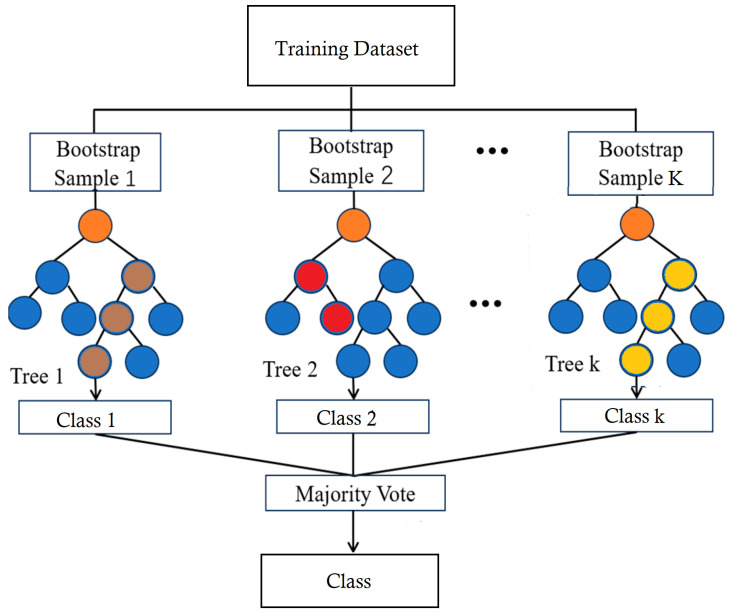
Visualization of an RF classifier.

Given that a random forest comprises *T* decision trees, the trees’ prediction outputs are denoted by $Q_{i}\left(x\right)$. Therefore, each bootstrap sample produces DT models, as shown in Figure 14. Based on color-coded nodes in the figure, orange nodes are root nodes that start the splitting process based on the most significant feature. Blue nodes are intermediate splits, and red nodes reflect unique decisions from bootstrap sampling. Yellow and brown nodes are leaf nodes where final class decisions are made. Finally, the class decisions are made, contributing to the majority vote in the RF. An increasing number of trees causes a random forest’s generalization error to converge to a limit. The individual trees’ strengths and correlations with one another impact this error.

#### 2.2.8. Artificial Neural Networks (ANNs)

The architecture and operation of the human brain inspire computational models known as Artificial Neural Networks (ANNs). ANNs are an effective machine learning technique with several applications, such as pattern recognition, classification, and regression, due to their ability to learn complex patterns and relationships and their flexibility in handling various data types.

**Figure 15:**
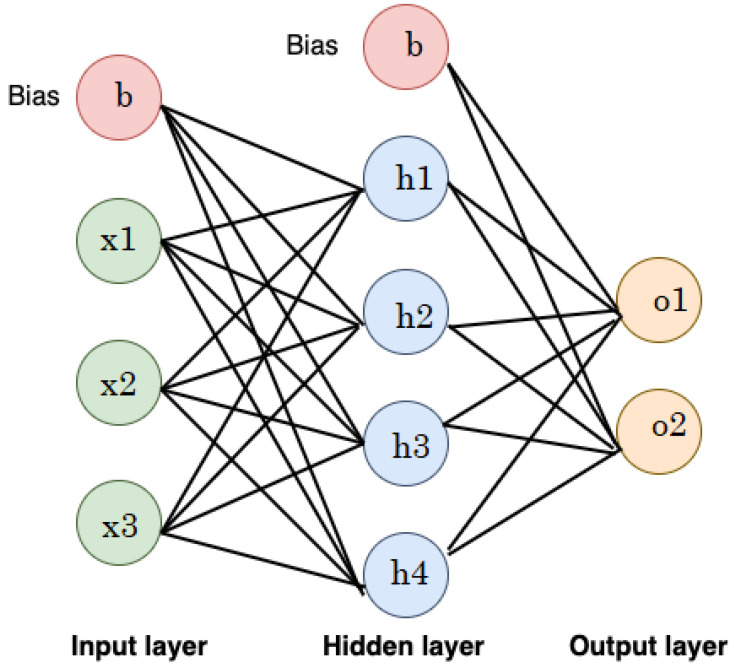
An illustrative example of a feedforward NN.

The general architecture of an ANN consists of multiple layers (input, hidden, and output layers), known as a multi-layer perception (MLP). The output of one layer feeds into the next layer sequentially. Neurons in the input layer reflect the features fed into the model. The hidden layer’s neurons vary according to the task at hand. Ultimately, the number of classes in the dataset is directly proportional to the number of neurons in the output layer. The output (*y*) is mathematically specified as a weighted sum of inputs processed by an activation function (*f*) (e.g., sigmoid or ReLU) based on Equation (10).

(10) $$y=f\left(\sum_{i=1}^{n}w_{i}x_{i}+b\right)$$

where $w_{i}$ and $x_{i}$ correspond to the weight and input, respectively; *b* is the bias of the ANN; and *n* is the number of neurons. Figure 15 shows an illustrative example of a feedforward NN consisting of an input layer (3 neurons), hidden layer (4 neurons), and output layer (2 neurons). The neurons in the input layer represent the features, while the neurons in the output layer represent the output class.

### 2.3. COPD Diagnosis Model and Performance Metrics

**Figure 16:**
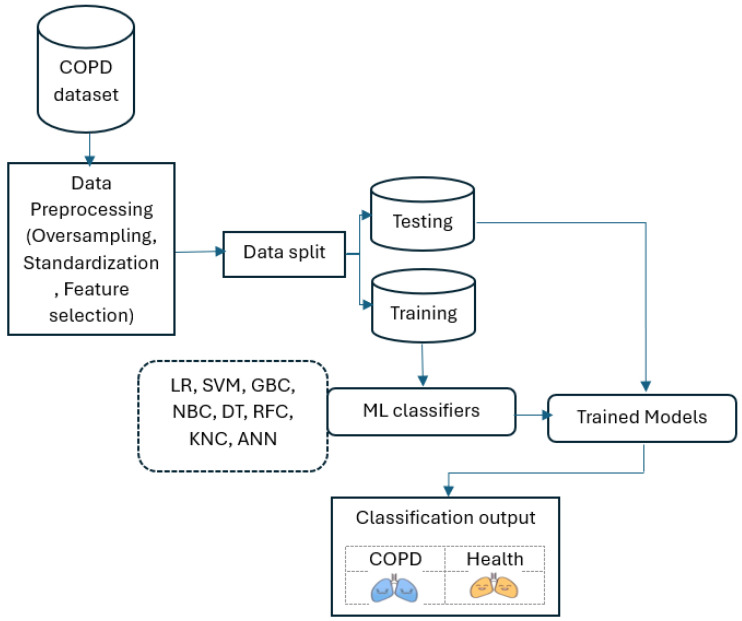
COPD diagnosis utilizing various machine learning techniques.

Figure 16 shows the chronic obstructive pulmonary disease diagnosis process utilizing various machine learning techniques.

**Figure 17:**
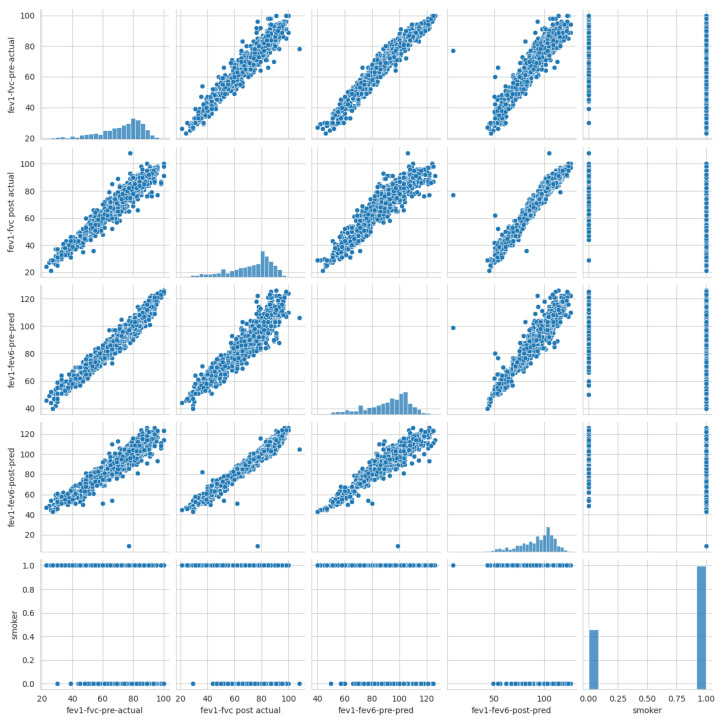
Visualization of significant features associated with COPD.

The figure illustrates a machine learning (ML) workflow for diagnosing chronic obstructive pulmonary disease (COPD), which is utilized in this study. Bellow is a breakdown of the key components:

- COPD Dataset: This represents the data collection used to train and test the ML models. It likely contains various features extracted from medical records, such as patient demographics, symptoms, and lung function tests.
- Data Preprocessing: In this study, data preprocessing involved three key steps to ensure the quality and reliability of the machine learning process. First, one of the simplest and most effective oversampling techniques was applied, which is “RandomOverSampler”, to address class imbalance and improve the representation of minority classes as illustrated in Algorithm 1. As a result of oversampling, the dataset comprised 2030 data points. Second, data scaling was performed using “StandardScaler” to normalize feature values and eliminate biases caused by differing scales. It transforms data by centering around zero (subtracting the mean) and scaling to a standard deviation of one. Finally, feature selection methods were employed to identify and retain the most relevant predictors, reducing model complexity and enhancing efficiency. In this study, five features were selected (fev1-fvc-pre-actual, fev1-fvc post-actual, fev1-fev6-pre-pred, fev1-fev6-post-pred, and smoker) based on correlation coefficients, as shown in Figure 3. The correlation of the selected significant features with the COPD class label is shown in Figure 17. These preprocessing steps were crucial in preparing the dataset for robust analysis and ensuring model performance.

**Algorithm 1:** Balancing the Dataset Using Oversampling  1: **Input:** Dataset *D* with majority class $D_{\mathrm{maj}}$ and minority class $D_{\min}$
  2: **Output:** Balanced dataset $D_{\mathrm{balanced}}$
  3: Identify the minority class $D_{\min}$ and majority class $D_{\mathrm{maj}}$
  4: **While** Size($D_{\min}$) < Size($D_{\mathrm{maj}}$) **do**
  5: Randomly select samples from $D_{\min}$
  6: Duplicate the selected samples
  7: Add the duplicated samples to $D_{\min}$
  8: **End While**
  9: Combine $D_{\min}$ and $D_{\mathrm{maj}}$ to form $D_{\mathrm{balanced}}$
  10: **Return** 
$D_{\mathrm{balanced}}$

- Data Split: The dataset utilized in this study is divided into two segments: training (75%) and testing (25%). A total 1522 data points were allocated to the training set, and 508 were designated for testing. The training set teaches the ML models to recognize patterns and relationships within the data, while the testing set is used to evaluate the models’ performance on unseen data.
- ML Classifiers: Various ML algorithms were employed, including LR, SVM, GBC, NBC, DT, RFC, KNC, and ANN.
- Trained Models: Each ML classifier was trained on the training dataset, resulting in a set of trained models. These models learned to differentiate between patients with COPD and those without.
- Classification Output: The trained models were applied to the testing dataset to predict whether each patient had COPD or was healthy. The output is a classification label for each patient.

The figure demonstrates a standard approach to developing ML models for disease diagnosis. By comparing the performance of different ML algorithms on the same dataset, researchers can identify the most effective methods for diagnosing COPD.

Furthermore, a set of performance metrics was used to measure the effectiveness of each model. These performance metrics include accuracy, precision, recall, and F1 score. All these measures are based on four factors, which are correctly classified as COPD (+COPD) or healthy (+Healthy) patients and incorrectly classified as COPD (-COPD) or healthy patients (-Healthy).

- Accuracy (Acc) refers to the ability to correctly predict whether a patient has the disease. It is calculated as the proportion of correct predictions (both true positives and true negatives, which are identified as the total of +COPD and +Healthy) out of the total number of predictions (i.e., TotalPredications= (+COPD) + (-COPD) + (+Healthy) + (-Healthy)).
  (11) $$Acc=\frac{\left(+\mathrm{COPD})+(+\mathrm{Healthy}\right)}{TotalPredications}$$
- Precision (Pre) indicates how often the model is correct when it predicts a positive outcome.
  (12) $$Pre=\frac{+\mathrm{COPD}}{\left(+\mathrm{COPD})+(-\mathrm{Healthy}\right)}$$
- Recall (Rec) shows how sensitive the model is to positive instances.
  (13) $$Rec=\frac{+\mathrm{COPD}}{\left(+\mathrm{COPD})+(-\mathrm{COPD}\right)}$$
- The F score (Fscore) reflects a model’s ability to both accurately identify positive cases and avoid false positives.
  (14) $$F1=2\cdot \frac{Pre\cdot Rec}{Pre+Rec}$$

**Figure 18:**
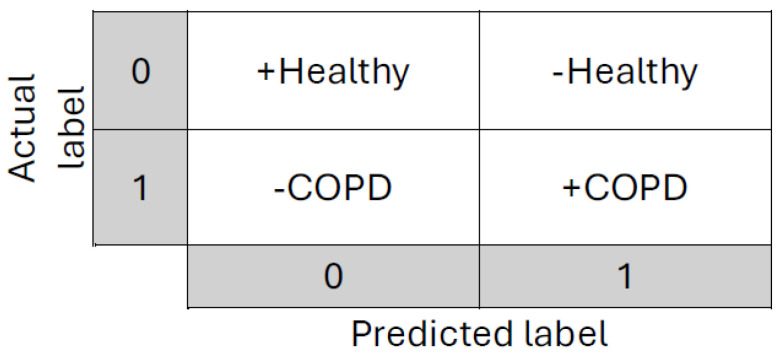
Visual representation of a binary confusion matrix for classification of COPD patients.

Furthermore, the binary confusion matrix can identify how many patients are correctly classified in each class. In the case of COPD patients, the predicted outcome is (+COPD), indicating that they have the disease, or (+Healthy), indicating that individuals are considered healthy. In contrast, (-Healthy) occurs when a healthy individual is misclassified as a COPD patient, and (-COPD) arises when a COPD patient is misidentified as healthy. A visual representation in the form of a confusion matrix is shown in Figure 18.

## 3. Experimental Results

Various machine learning classifiers are used to diagnose COPD, including LR, SVM, GBC, GNB, RFC, KNC, ANN, and DT. Each method has unique advantages and drawbacks, and selecting the most suitable approach hinges on factors such as the problem’s characteristics, dataset size, data quality, and available computational resources. We developed and executed these models in the Google Colab environment. Google Colab is a cloud-based platform that efficiently executes machine learning workflows. It offers an Python-based climate pre-installed with popular libraries such as Scikit-learn.

The results of the classification models used for the training and testing sets are displayed in Table 1 regarding various performance metrics. The best results are presented in Bold. The RFC is the best-performing model in training across all metrics, with an accuracy equal to 0.8206. Also, it consistently achieves the highest F score in testing. The GBC performs well on training and test sets, demonstrating its generalization ability. While both SVM and DT achieved similar levels of accuracy on the training data, SVM’s superior performance on the test set, with a 0.29% increase in accuracy, indicates its more potent ability to generalize to unseen data. LR exhibits moderate performance on the training and test sets, while KNC shows a significant discrepancy between training and test performance. Both GNB and ANN achieved the lowest accuracy in training and testing.

**Figure 19:**
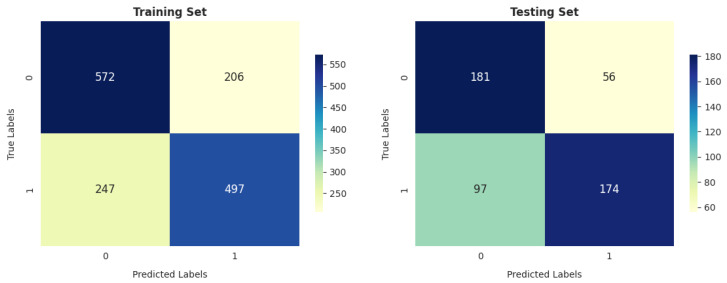
LR confusion matrix.

**Figure 20:**
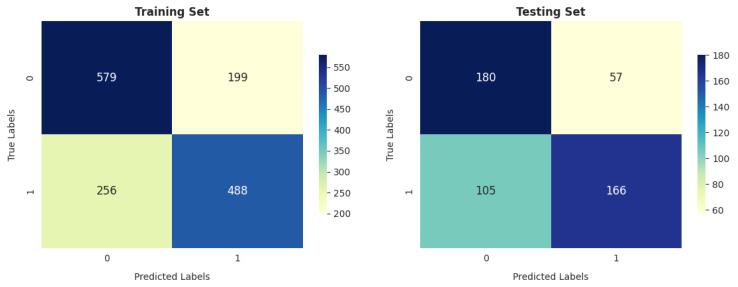
SVM confusion matrix.

**Figure 21:**
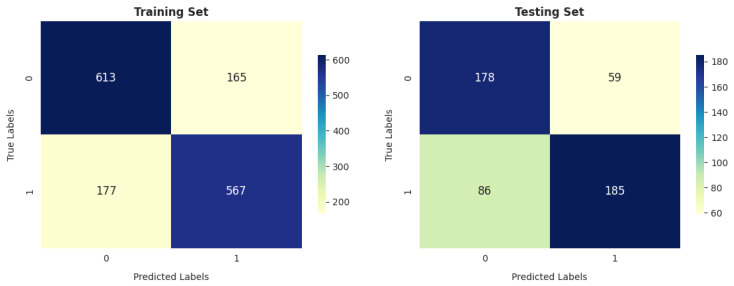
GBC confusion matrix.

**Figure 22:**
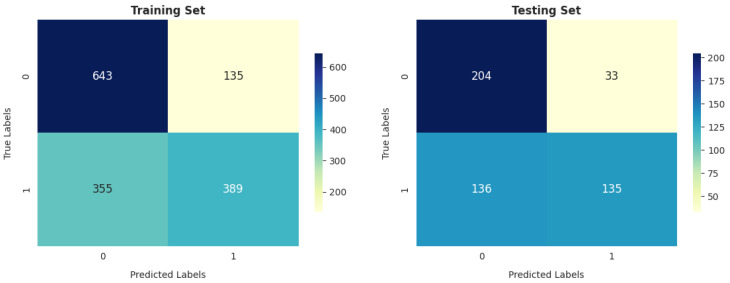
GNB confusion matrix.

**Figure 23:**
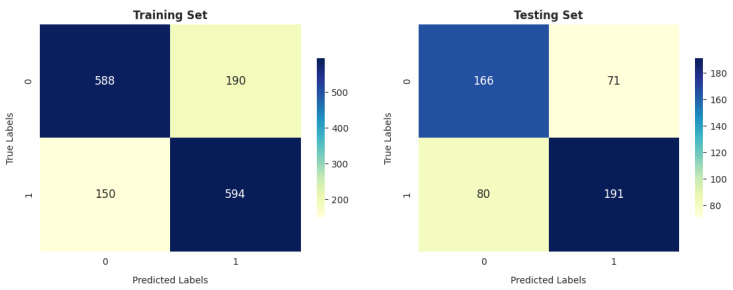
KNC confusion matrix.

**Figure 24:**
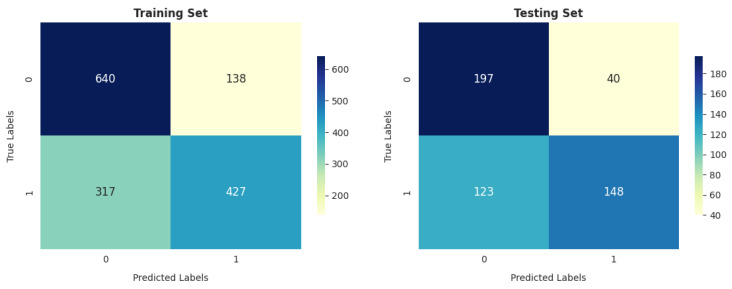
DT confusion matrix.

**Figure 25:**
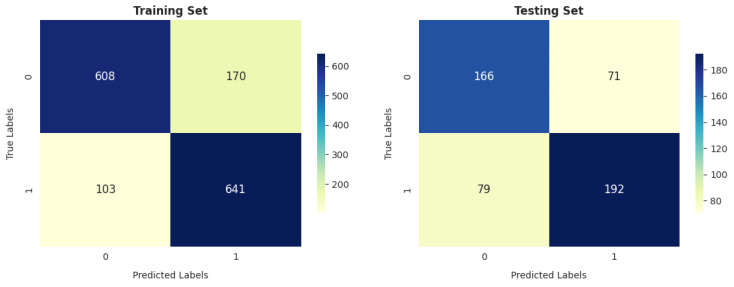
RFC confusion matrix.

**Figure 26:**
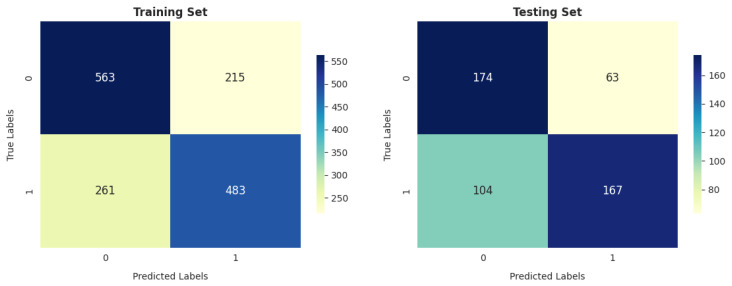
ANN confusion matrix.

Moreover, Figure 19, Figure 20, Figure 21, Figure 22, Figure 23, Figure 24, Figure 25 and Figure 26 present the confusion matrices corresponding to the various methodologies utilized on the training and testing datasets. According to Figure 19, the LR model performs better in training than testing, with a higher number of correct classifications (1069) compared to incorrect classifications (453) out of 1522 in training and a higher number correct classifications (355) compared to incorrect classifications (153) out of 508 in testing.

SVM performs better in reducing the number of false-positive cases (i.e., -healthy): to 199 and 57 in training and testing, respectively, as shown in Figure 20.

According to Figure 21, the gradient boosting classifier achieved better results than SVM and LR in identifying the number of correct cases on both training and testing data. The number of correctly classified COPD and healthy patients was 1180 (77.53%) and 363 (71.46%) in training and testing, respectively.

The GNB model achieved a lower total number of (+COPD) and (+Health) classifications, equal to 1032 in training, as shown in Figure 22, compared with LR, SVM, and GBC. It also achieved a higher total number of incorrectly classified cases ((-COPD) and (-Health)), equal to 490 in training and 169 in testing.

Figure 23 shows that the number of positive cases predicted as COPD was 594 (39.03%) and those predicted as healthy was 588 (38.63%), while the number of misclassifications of COPD was 150 (9.86%) and that of misclassifications of healthy patients was 190 (12.48%) on training data. In testing, the KNC incorrectly identified 151 out of 508 (29.72%) cases as COPD and healthy.

The DT and SVM models show similar trends in terms of performance on the training and testing sets, as shown in Figure 20 and Figure 24, with 1067 correctly identified instances and 455 incorrectly identified instances in training.

As shown in Figure 25, the RFC achieved the highest number of correct instances, equal to 1249, and the fewest incorrect cases, equal to 273 out of 1522, among all the compared algorithms. However, in testing, the GBC reached a total number of correctly classified instances better than that of the RFC.

The ANN model’s performance is comparable to that of GNB on the training and testing sets, especially in terms of the total number of correctly classified instances (1046 out of 1522 and 341 out of 508 in training and testing, respectively).

**Figure 27:**
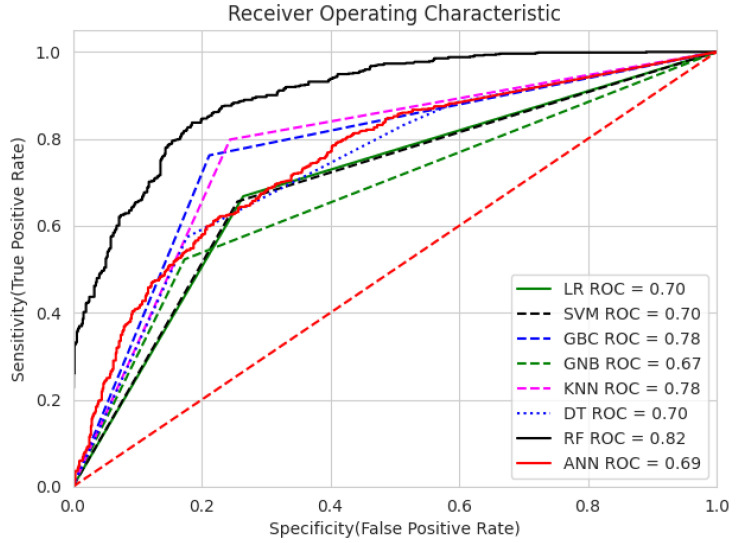
The ROC curves for various ML techniques.

**Figure 28:**
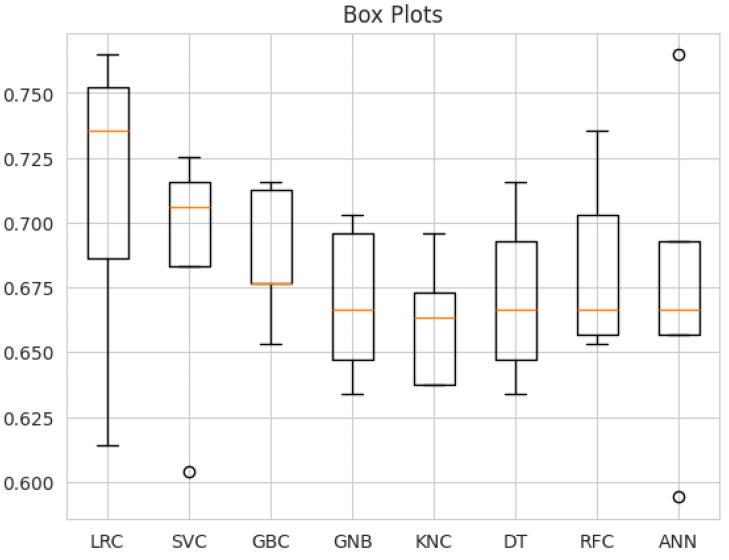
Box-and-whisker plot for the used techniques.

Furthermore, Figure 27 and Figure 28 show the Receiver Operating Characteristic curves (ROC curve) and box plots, respectively, for the utilized machine learning classifiers. RFC has achieved the best ROC value of 0.82 compared to others, where a greater AUC indicates better class discrimination. Other models, such as the gradient boosting classifier (GBC) and KNN, also demonstrate reasonable performance, with AUC values close to 0.78. Generally, a model with a curve closer to the plot’s top-left corner indicates better performance.

The box plot shown in Figure 28 illustrates the performance of various machine learning models, including LRC, SVC, GBC, GNB, KNC, DT, RFC, and ANN, based on accuracy. The LRC model has the highest median accuracy (0.73) but shows significant variability, as indicated by its wide interquartile range (IQR) and outliers below 0.60. SVC exhibits more consistent results, with a narrower IQR and a median around 0.70, although it still has an outlier at 0.60. The GBC and GNB models have moderate IQRs, reflecting fairly consistent performance, with median accuracies close to 0.70. KNC and DT show somewhat wider IQRs, indicating more variability in their performance, with medians around 0.67. The RFC has a similarly wide IQR, indicating significant performance variability despite a median of 0.69. Finally, the ANN model, with a median accuracy of approximately 0.68, shows a narrower IQR, suggesting stable performance but also some outliers above and below 0.70. Overall, the RFC demonstrates strong performance, with a relatively high median accuracy. However, its variability suggests that while the model can achieve excellent results in some cases, it lacks the consistency observed in models like SVC an ANN. Thus, the RFC may be a suitable choice when peak performance is prioritized.

The analysis suggests that the RFC performs best on the training dataset. Meanwhile, the RFC demonstrates strong performance on the testing dataset in terms of recall and F score, which highlight its ability to correctly identify positive instances and maintain precision.

## 4. Discussion and Analysis of the Results

This study utilized various ML classifiers to diagnose COPD. As shown in Table 1, our results revealed the following in comparison with some previous studies:

- Our analysis of 1603 patient records revealed that the random forest classifier (RFC) outperformed other models, achieving the highest accuracy, reaching up to 82.06%; precision; recall; and F1 score on the training dataset. It obtained a perfect score and achieved a good balance of false positives and false negatives. This is consistent with the findings of previous research [16], which analyzed 6883 patients from the United Kingdom and the random forest model was found to outperform the others, achieving an impressive accuracy rate of up to 92%. Furthermore, its performance on the testing dataset remained entirely satisfactory, as it achieved an accuracy of 70.47% and the highest F score of 71.91%.
- The gradient boosting classifier (GBC) also demonstrates strong performance on the training dataset, achieving 77.53%, 77.46%, 76.76%, and 76.83% scores for accuracy, precision, recall, and F score, respectively. When tested on the validation dataset, it maintains solid performance, with an accuracy of 71.46%. This accuracy surpasses that of other models.
- The decision tree classifier (DTC) and support vector machine (SVM) exhibit a reasonable accuracy of 70.11% on the training dataset. However, the performance of DT on the testing dataset is somewhat lower, at 67.91%.
- The K-nearest neighbors classifier (KNC) shows strong training results, with an accuracy of 77.66%, precision of 75.77%, recall of 79.84%, and F score of 77.75%. The high recall suggests the model is sensitive to the positive class. While there is a slight drop in testing accuracy compared to training, the model still performs well in generalization.
- Logistic regression (LR) demonstrates decent performance, with a training accuracy of 70.24% and testing accuracy of 69.88%, indicating its ability to generalize reasonably well. It shows good precision on both training (70.70%) and testing (75.65%) datasets, suggesting that it is reliable in avoiding false positives. However, its recall is relatively lower, with 66.80% on the training set and 64.21% on the test set, meaning it misses a significant portion of positive cases. This results in an F score of 68.69% for training and 69.46% for testing, reflecting a moderate balance between precision and recall. While LR is a solid performer overall, it falls short of more complex models like RFC and KNC regarding recall and overall performance.
- Meng et al. [18] employed five common machine learning algorithms (naïve Bayes, KNC, DT, RF, and LR), along with several nonlinear predictors, to characterize PM2.5 pollution-sensitive COPD patients (327 patients), and their results showed that all classifiers yielded similar AUC values. The sensitivities of naïve Bayes, KNC, DT, RF, and LR were 61%, 50%, 46%, 48%, and 39%, respectively. Among these, naïve Bayes outperformed the other algorithms, achieving the highest AUC value of 0.7673. In contrast, our results reveal that the RFC achieved the highest AUC value of 0.82, and the sensitivities of RF, KNC, DT, and LR were 70.85%, 70.48%, 54.61%, and 64.21%, respectively, on the testing data.
- GNB achieves 74.24% precision, which is strong, but its recall is significantly lower (52.28%), leading to a relatively low F score of 61.36% on training data. On the test data, GNB’s performance is even weaker, with 66.73% accuracy and 80.36% precision, as well as a much lower recall of 49.82%. This makes it less suitable compared to the models with more well-balanced performance, such as the RFC and KNC.
- The artificial neural network (ANN) shows moderate performance across both training and testing datasets. Its recall value dropped from 64.92% (in training) to 64.92% (in testing), indicating that it misses a considerable number of positive instances on the test data. The authors of [26] reported that a multilayer ANN achieved better results than SVM when considering 695 patient records.

Moreover, in terms of discrimination of two classes (COPD/healthy) during training, the RFC achieved superior results in correctly classifying both classes. However, the KNC achieved the fewest misclassified instances (340 out of 1522), followed by GBC (342 out of 1522) in training. Although the GBC achieved the highest accuracy in testing, the RF was superior in recall by 3.65% and F1 score by 0.09%. GNB achieved the fewest correctly classified instances (339) compared to others. Our experiments revealed that RFC is the top-performing model, delivering strong results on both the training and test sets. It stands out for achieving the highest ROC value, as well as its robustness in achieving limited variability in performance across different iterations.

## 5. Statistical Test

Friedman’s non-parametric statistical test was utilized to thoroughly evaluate the classification algorithms’ performance. This approach was designed to determine which classification method achieved statistically significant superiority over the others. Table 2 presents the mean rankings derived from Friedman’s test, comparing the competing classification techniques regarding classification accuracy, precision, recall, and F-score rates.

Table 2 demonstrates that better performance is associated with lower mean rankings. Several *p*-values derived using Friedman’s test and reported in the same table are below the significance threshold of α=0.5. The RFC consistently ranks highest across all metrics, with the lowest mean ranks for accuracy (1.5), recall (1.0), and F1 score (1.0). This indicates that the RFC is the most effective classifier among the competitors. The GBC also performs well, achieving the second-best rankings for most metrics (accuracy: 2.0; F1 score: 2.5). However, the KNC exhibits strong performance in recall (2.0) and good results in accuracy and F1 score (2.5), showing it as a competitive alternative, while DT performs moderately well in precision (3.0) but poorly in recall (7.0) and F1 score (7.0), indicating variability in its effectiveness. On the contrary, the GNB and ANN classifiers exhibit the poorest performance among the models. GNB consistently ranks lowest across all metrics, and ANN shows high variability and subpar results, particularly in precision. Furthermore, The *p*-values for accuracy (0.063) and recall (0.054) are close to the typical significance threshold of 0.05, suggesting a potential trend toward statistically significant differences among the classifiers for these metrics. Conversely, *p*-values for precision (0.333) suggest no statistically significant differences in this metric, reflecting similar performance among the classifiers and borderline significant differences for the F1 score (0.054).

Holm’s procedure was subsequently applied as a post hoc statistical method to demonstrate the extent of the differences between the control classifier and its competitors. Based on the results of Friedman’s test, the control classification method was found to outperform the others across all evaluation metrics. The statistical outcomes from Holm’s analysis are detailed in Table 3. Friedman’s rank of the control classifier is represented by $R_{0}$, the rank of the *i*th classifier is $R^{i}$, the effect size (ES) reflects the impact of the control classification method on the *i*th classifier, and *z* indicates the statistical difference between the two classification methods.

Holm’s test evaluated the competing classification systems by rejecting hypotheses with *p*-values less than or equal to 0.00714286 for accuracy and precision and 0.00833333 for recall and F1 score. The RFC significantly outperforms GNB based on accuracy, while no significant differences are observed relative to other classifiers. In terms of precision, the GBC demonstrates a statistically significant advantage over ANN, but differences relative to other classifiers are not substantial. The RFC shows strong recall performance, particularly against GNB and DT, while other classifiers are statistically similar. Similar to F1 score, the RFC significantly outperforms GNB and DT, while no significant differences are found between the RFC and other classifiers. Overall, the RFC and GBC emerge as the strongest classifiers, consistently ranking at the top across metrics, whereas GNB exhibits significant underperformance. ANN shows potential but underperforms in precision when compared to the GBC. These findings highlight the robustness of the RFC as a trustworthy classification technique with appropriate behavior.

## 6. Conclusions and Future Directions

Given the significant impact of chronic obstructive pulmonary disease (COPD) on the adult population and the challenges posed by expensive and logistically complex traditional diagnostic methods, various machine learning techniques were utilized in this study. Among these models, the random forest classifier emerged as the top performer, boasting an accuracy and F score of 70.47% and 71.91%, respectively. Furthermore, the ROC curve produced a significance level of 0.74. This study achieved a classification accuracy that aligns with other relevant research in the field. Furthermore, the study leveraged readily obtainable physical parameters, offering physicians and patients a more practical diagnostic approach. Future studies might focus on additional subjects and more generalizable models. The feasibility of COPD monitoring also requires more investigation.

## Figures and Tables

**Table 1:** Calculated performance metrics of various ML models.

Model	Train				Test			
	Acc	Pre	Rec	Fscore	Acc	Pre	Rec	Fscore
LR	0.7024	0.7070	0.6680	0.6869	0.6988	0.7565	0.6421	0.6946
SVM	0.7011	0.7103	0.6559	0.6820	0.6811	0.7444	0.6125	0.6721
GBC	0.7753	0.7746	0.7621	0.7683	0.7146	0.7582	0.6827	0.7184
GNB	0.6781	0.7424	0.5228	0.6136	0.6673	0.8036	0.4982	0.6150
KNC	0.7766	0.7577	0.7984	0.7775	0.7028	0.7290	0.7048	0.7167
DT	0.7011	0.7558	0.5739	0.6524	0.6791	0.7872	0.5461	0.6449
RFC	**0.8206**	**0.7904**	**0.8616**	**0.8244**	0.7047	0.7300	**0.7085**	**0.7191**
ANN	0.6873	0.6920	0.6492	0.6699	0.6713	0.7261	0.6162	0.6667

**Table 2:** Mean ranking outcomes of all competing classifications using Friedman’s statistical test.

Classifier	Accuracy	Precision	Recall	F1 Score
LR	4.0	5.5	4.0	4.0
SVM	5.25	5.5	5.5	5.0
GBC	2.0	2.5	3.0	2.5
GNB	8.0	3.0	8.0	8.0
kNC	2.5	5.0	2.0	2.5
DT	5.75	3.0	7.0	7.0
RFC	1.5	3.5	1.0	1.0
ANN	7.0	8.0.0	5.5	6.0
*p*-value	0.0634819	0.3325939	0.0542263	0.0542263

**Table 3:** Holm’s test results between the classification methods.

Accuracy (RF is the control classifier)					
i	Algorithm	$z=\frac{\left(R_{0}-R^{i}\right)}{SE}$	*p*-value	α ÷ *i*	Hypothesis
7	GNB	2.65361389	0.00796349	0.00714286	Rejected
6	ANN	2.24536560	0.02474467	0.00833333	Not Rejected
5	DT	1.73505523	0.08273102	0.01	Not Rejected
4	SVM	1.53093109	0.12578642	0.0125	Not Rejected
3	LR	1.02062073	0.30743417	0.01666667	Not Rejected
2	KNC	0.40824829	0.68309140	0.025	Not Rejected
1	GBC	0.20412415	0.83825649	0.05	Not Rejected
Precision (GBC is the control classifier)					
i	Algorithm	$z=\frac{\left(R_{0}-R^{i}\right)}{SE}$	*p*-value	α ÷ *i*	Hypothesis
7	ANN	2.24536560	0.02474467	0.00714286	Rejected
6	LR	1.22474487	0.22067136	0.00833333	Not Rejected
5	SVM	1.22474487	0.22067136	0.01	Not Rejected
4	KNC	1.02062073	0.30743417	0.0125	Not Rejected
3	RFC	0.40824829	0.68309140	0.01666667	Not Rejected
2	GNB	0.20412415	0.83825649	0.025	Not Rejected
1	DT	0.20412415	0.83825649	0.05	Not Rejected
Recall (RFC is the control classifier)					
i	Algorithm	$z=\frac{\left(R_{0}-R^{i}\right)}{SE}$	*p*-value	α ÷ *i*	Hypothesis
7	GNB	2.85773803	0.00426672	0.00714286	Rejected
6	DT	2.44948974	0.01430588	0.00833333	Rejected
5	SVM	1.83711731	0.06619258	0.01	Not Rejected
4	ANN	1.83711731	0.06619258	0.0125	Not Rejected
3	LR	1.22474487	0.22067136	0.01666667	Not Rejected
2	GBC	0.81649658	0.41421618	0.025	Not Rejected
1	KNC	0.40824829	0.68309140	0.05	Not Rejected
F1 score (RFC is the control classifier)					
i	Algorithm	$z=\frac{\left(R_{0}-R^{i}\right)}{SE}$	*p*-value	α ÷ *i*	Hypothesis
7	GNB	2.85773803	0.004266725	0.00714286	Rejected
6	DT	2.44948974	0.014305878	0.00833333	Rejected
5	ANN	2.04124145	0.041226833	0.01	Not Rejected
4	SVM	1.63299316	0.102470435	0.0125	Not Rejected
3	LR	1.22474487	0.220671362	0.01666667	Not Rejected
2	GBC	0.61237244	0.540291375	0.025	Not Rejected
1	KNC	0.61237244	0.540291375	0.05	Not Rejected

## Data Availability

The data presented in this study are available upon request from the corresponding author subject to appropriate privacy safeguards and approval.
